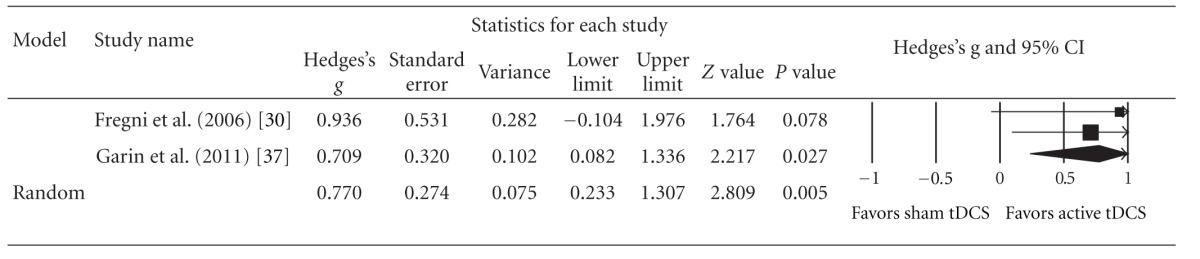# Transcranial Direct Current Stimulation in Tinnitus Patients: A Systemic Review and Meta-Analysis

**DOI:** 10.1100/2012/427941

**Published:** 2012-10-17

**Authors:** Jae-Jin Song, Sven Vanneste, Paul Van de Heyning, Dirk De Ridder

**Affiliations:** ^1^Brain, TRI & Department of Neurosurgery, University Hospital Antwerp, Wilrijkstraat 10, 2650 Edegem, Belgium; ^2^Department of Otorhinolaryngology-Head and Neck Surgery, Seoul National University Hospital, Seoul 110-744, Republic of Korea; ^3^Department of Translational Neuroscience, Faculty of Medicine, University of Antwerp, 2650 Edegem, Belgium; ^4^Brain, TRI & ENT, University Hospital Antwerp, Wilrijkstraat 10, 2650 Edegem, Belgium; ^5^Department of Surgical Sciences, Dunedin School of Medicine, University of Otago, Dunedin 9016, New Zealand

## Abstract

Although transcranial direct current stimulation (tDCS) has already been used to manage tinnitus patients, paucity of reports and variations in protocols preclude a comprehensive understanding. Hence, we conducted a meta-analysis based on systemic review to assess effectiveness of tDCS in tinnitus management and to compare stimulation parameters. PubMed was searched for tDCS studies in tinnitus. For randomized controlled trials (RCTs), a meta-analysis was performed. A total of 17 studies were identified and 6 of them were included in the systemic review and 2 RCTs were included in the meta-analysis. Overall 39.5% responded to active tDCS with a mean tinnitus intensity reduction of 13.5%. Additionally, left temporal area (LTA) and bifrontal tDCS indicated comparable results. Active tDCS was found to be more effective than sham tDCS for tinnitus intensity reduction (Hedges' *g* = .77, 95% confidence interval 0.23–1.31). The efficacy of tDCS in tinnitus could not be fully confirmed by the current study because of the limited number of studies, but all studies included in the current systemic review and meta-analysis demonstrated significant tinnitus intensity improvement. Therefore, tDCS may be a promising tool for tinnitus management. Future RCTs in a large series regarding the efficacy as well as the comparison between LTA- and bifrontal tDCS are recommended.

## 1. Introduction

Subjective tinnitus, a phantom sound perception in the absence of an identifiable objective, external sound source [1], afflicts 5%–21% of adults at some point in their lifetime and increases in people exposed to work-related [2] or leisure-related [2, 3] noise exposure. This high prevalence has been attributed to the free energy principle, in which the reduced auditory input results in Bayesian frequency specific updating in an attempt to reduce environmental auditory uncertainty associated with this auditory deafferentation [4]. Although often not fully appreciated by the general public, tinnitus is one of the most debilitating audiological disorders and affects almost all aspects of daily life [5, 6], lowering the quality of life in 1% of the total population [7–9]. Cognitive impairments, sleep disturbances, negative emotions, and other psychiatric comorbidities such as depression associated with tinnitus are especially bothersome for patients and their families [10, 11]. 

Although numerous management disciplines including pharmacological and nonpharmacological treatments have been introduced, evidence for a uniformly successful treatment that can eliminate tinnitus is lacking [12]. Because the initial diagnosis and evaluation of treatment effects cannot be objectified easily, the treatment goals are aimed at symptomatic relief relying on patients' subjective symptom reports in the majority of cases. The absence of standardized single gold-standard treatment for tinnitus thus necessitates combinations of treatment strategies or developments of novel treatment modalities.

With the development of the idea that the unified tinnitus percept is an emergent network property resulting from activity in multiple, parallel, partially overlapping but separable networks [13] encompassing both auditory and nonauditory brain areas [14, 15], new treatments are being developed, including both pharmacological [16] and neuromodulatory approaches [17]. 

Over the last decade, noninvasive neuromodulations such as transcranial magnetic stimulation (TMS), transcranial direct current stimulation (tDCS), transcutaneous electrical nerve stimulation, and neurofeedback have been used, as well as invasive neuromodulation techniques. These include implantable cortical electrodes on the auditory and the dorsolateral prefrontal cortex (DLPFC), as well as subcutaneous occipital nerve stimulation, and deep brain stimulation [18], especially for cases of intractable tinnitus. 

Of these neuromodulation methods, tDCS might become a clinically useful noninvasive neuromodulation technique for tinnitus suppression due to its low cost, easy, painless application, and its longer residual inhibition than TMS. tDCS delivers low direct currents (0.5–2 mA) via scalp electrodes to the cerebral cortex that result in the modulation of cortical excitability for variable periods outlasting the stimulation period [19]. A part of this current is shunted through the scalp and the rest flows into the cerebral cortex, thereby increasing or decreasing cortical excitability in the brain regions to which it is applied depending on the polarity of the stimulation [20]. Currently, tDCS is usually applied through 2 surface electrodes, one serving as an anode and the other as a cathode. Anodal tDCS typically has an excitatory effect on the underlying cerebral cortex by depolarizing neurons, while cathodal tDCS decreases cortical excitability by induced hyperpolarization [21]. This excitability changing effect of tDCS is typically maintained for an hour or longer after a single session of sufficiently long stimulation duration [21–24]. 

tDCS has been applied for treating major depression [25–27] and chronic pain [28, 29] with relatively promising outcomes. Also, it has been used to manage tinnitus patients since the first application of tDCS for treating tinnitus by Fregni et al. [30]. However, paucity of accumulated treatment results and study-to-study variations in stimulation protocols preclude physicians from achieving a comprehensive understanding of the therapeutic value of tDCS for tinnitus. Hence, by conducting the current meta-analysis based on systemic review on treatment results of tDCS in tinnitus, we aimed at assessing effectiveness of tDCS for tinnitus reduction and identifying the most desirable combination of stimulation parameters. 

## 2. Methods and Materials 

### 2.1. Data Sources

To identify all studies available, PubMed searches on tDCS studies on tinnitus according to PRISMA (Preferred Reporting Items for Systematic reviews and Meta-Analyses) guidelines [31] were conducted. Keywords used in this search were: “transcranial direct current stimulation,” “tDCS,” and “tinnitus” with activated limit to article types other than review, human species, and English language. In this way, open-label studies and randomized controlled trials (RCTs) on tDCS in tinnitus patients were identified. The search was performed in August 2012, with a start date of January 1, 1998. The start date was selected as the date of the first study performed with contemporary stimulation parameters [32] and has also been employed in recent reviews of tDCS [33, 34].

### 2.2. Study Selection

All identified studies were examined by 2 authors (J. J. Song and S. Vanneste) independently. The inclusion criteria for the current meta-analysis were that studies (1) published in a peer-reviewed journal, (2) reporting on tDCS in the management of tinnitus patients, (3) dealing with original data of open-label or RCT with tinnitus loudness as the outcome measure, (4) performed by randomized parallel or crossover design, with sham control, and (5) where both participants and raters had to be unaware of treatment condition. Percentage change in tinnitus intensity measure had to be either directly available or possible to derive from the publication by the data shown in tables or figures. In crossover trials, only data from studies with sufficient wash-out period (more than 2 weeks) between trials were used to avoid carry-over effects between trial stages.

### 2.3. Data Extraction and Analysis

For initial systemic review, the following data were extracted by an author (J. J. Song) in a structured fashion and then confirmed by another author (S. Vanneste): (1) study design, (2) patient characteristics (age, tinnitus laterality and tinnitus sound characteristics), (3) tDCS parameters (electrode placement, electrode size, current strength, duration of stimulation, duration of intermission between stimulations, number of treatment sessions, duration of wash-out period), and (4) results (percentage change in tinnitus loudness score, percentage of tDCS responders, any long-lasting beneficial effects). In case of missing or incomplete information, data were extracted from the figures and tables as much as possible. 

From the systemic review data, weighted means for the percentage change in tinnitus intensity and percentage of tDCS responders were calculated. Additionally, provided we thought it clinically relevant, and no important clinical and methodological heterogeneity was found, we summarized results in a meta-analysis. In this way, the efficacy of tDCS was explored by calculating random model effect sizes (Hedges' *g*) based on percentage change in tinnitus intensity in active and sham groups. Random-effects model is considered more conservative than a fixed-effect model, since it takes into account the variability between studies, thus leading to wider confidence intervals (CIs) [35]. 

The meta-analysis was undertaken using Comprehensive Meta-Analysis (CMA) Version 2 software (Biostat, Englewood, New Jersey, USA).

## 3. Results

### 3.1. Included Studies

**Figure 1:**
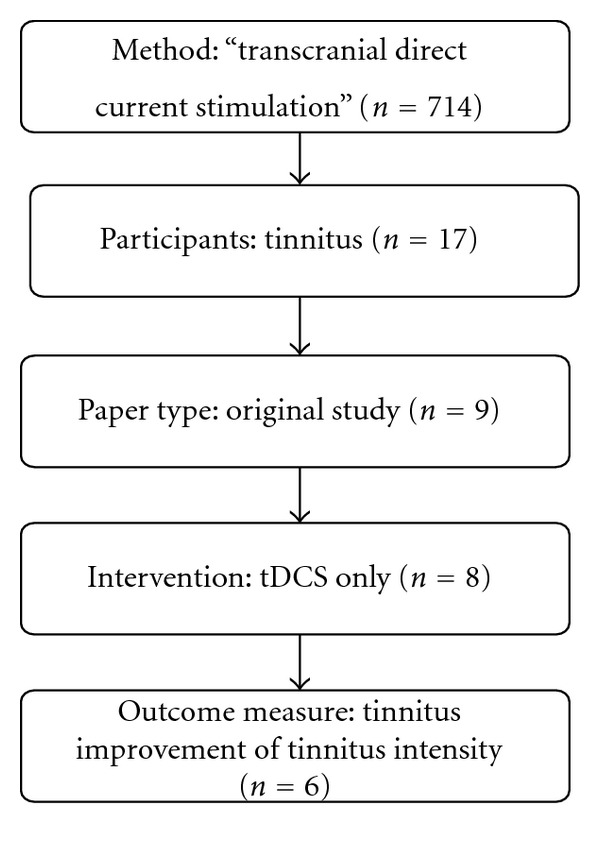
Illustration of the steps of study selection.

An initial search using keywords “transcranial direct current stimulation” or “tDCS” screened a total of 714 articles. Adding another keyword “tinnitus” sorted out 17 articles from 714. Of 17, six studies that met the above-mentioned inclusion criteria were included in the systematic review and are summarized in Table 1. Of the included studies, 3 were open-label trials and 3 were RCTs. The meta-analysis included 2 of 3 RCT studies and a total of 27 patients with tinnitus were randomized to active tDCS and 27 were randomized to sham tDCS. A flow diagram of the initial identification and attrition to the final selection of the studies is demonstrated in Figure 1.

### 3.2. Weighted Mean Percentage Responders to Active tDCS

The data of mean percentage responders to active tDCS were available in 2 RCT studies [30, 37] and 3 open label studies [38–40]. The weighted mean for percentage reduction of tinnitus intensity with active tDCS was 39.5% (RCT 17.6%, open label 11.6%), ranging from 29.9% [38] to 46.7% [39].

### 3.3. Weighted Mean Percentage Reduction of Tinnitus Intensity by tDCS

The data of mean percentage of tinnitus intensity reduction were available in 3 RCT studies [30, 36, 37] and 2 open label studies [38, 39]. The weighted mean for percentage reduction of tinnitus intensity with active tDCS was 13.5% (RCT 17.6%, open label 11.6%), ranging from 8.0% [38] to 30.4% [30].

### 3.4. A Comparison between Left Temporal Area (LTA) tDCS and Bifrontal tDCS with regard to Weighted Mean Percentage Responders

The data of mean percentage responders to active tDCS were available in 2 LTA tDCS studies (all RCTs) [30, 37] and 3 bifrontal tDCS studies (all open-label trials) [38–40]. The weighted mean percentage of responders to active LTA tDCS were 37.0% (range, 35%–42.9%), while that to active bifrontal tDCS was 40.2% (range, 29.9%–46.7%).

### 3.5. A Comparison between LTA tDCS and Bifrontal tDCS with regard to Weighted Mean Percentage Reduction of Tinnitus Intensity

The data of mean percentage of tinnitus intensity reduction were available in 2 LTA tDCS studies (all RCTs) [30, 37] and 3 bifrontal tDCS studies (1RCT and 2 open-label trials) [36, 38, 39]. The weighted mean percentage of tinnitus intensity reduction by active LTA tDCS was 14.6% (range, 9.1%–30.4%), while that by active bifrontal tDCS was 13.1% (range, 8.0%–27.8%).

### 3.6. A Comparison between Active tDCS and Sham tDCS with regard to Tinnitus Intensity Reduction

As aforementioned, only RCTs were adopted for this meta-analytic comparison between active tDCS and sham tDCS with regard to tinnitus intensity reduction. As a result, only 2 of 3 RCTs were eligible for this meta-analysis. The pooled estimate of effect size (Hedges' *g*) for the reduction of tinnitus intensity as indicated by percentage reductions in tinnitus intensity between active and sham tDCS was 0.77 [*Z* = 2.81,  *P* = 0.005, 95% CI 0.23–1.31], indicating a significant medium to large effect size (Table 2). 

## 4. Discussion

The current systemic review and meta-analysis indicate that overall 39.5% of the tinnitus patients responded to active tDCS with a mean tinnitus intensity reduction of 13.5%. Meanwhile, the comparison between LTA tDCS and bifrontal tDCS yields comparable results with regard to percent responders and percent reduction of tinnitus intensity. Additionally, although only 2 studies were included, meta-analysis showed that LTA tDCS was associated with a significantly better treatment outcome as compared with sham tDCS.

### 4.1. Response to tDCS in Patients with Tinnitus

Although a meta-analytic approach to the mean percentage of the responders and the amount of tinnitus intensity reduction was impossible due to limited number of studies, the current systemic review of 6 studies revealed a 39.5% weighted mean response to tDCS and a 13.5% weighted mean reduction rate of the intensity. 

TDCS has been used in treating other pathologies such as chronic pain or depression and meta-analytic approaches to reveal the treatment efficacy have recently been made. A recent systemic review and meta-analysis of 10 tDCS studies in the treatment of major depression has reported a weighted mean response rate of 19.8% to tDCS and weighted mean symptom severity reduction rate of 28.9% [33]. Another meta-analysis of 5 tDCS studies in the treatment of pain failed to reveal a significant difference between active and sham stimulations [41]. In this regard, our results of tDCS in patients with tinnitus are comparable to other meta-analysis results of tDCS on other pathologies. Considering that tDCS for tinnitus is generally very well tolerated without any significant adverse effects [42], this systemic review reconfirms that tDCS for tinnitus is a promising noninvasive neuromodulatory treatment option.

### 4.2. Stimulation Site: LTA versus Bifrontal

The weighted mean percentage of responders to active LTA and bifrontal tDCS were 37.0% and 40.2%, respectively. Additionally, the weighted mean percentage of tinnitus intensity reduction by active LTA and bifrontal tDCS were 14.6% and 13.1%, respectively. These very preliminary comparisons of the stimulation sites suggest that these 2 locations of active electrodes were comparably effective for tinnitus treatment.

The era of tDCS application to tinnitus patients has begun by Fregni et al. study that introduced anodal tDCS of the LTA resulting in a transient reduction of tinnitus, similar to 10-Hz TMS [30]. Another recent study utilized tDCS of the LTA and showed significant reduction of tinnitus intensity in a larger group of patients, especially reported longer-lasting effects for several days in some patients [37]. However, both studies found no effect on cathodal tDCS of the LTA with the anode on the contralateral supraorbital area. This may be attributed to the fact that cathodal tDCS is too weak to change ongoing cortical electrical activity [18]. Therefore, tDCS with longer duration and elevated current was proposed to obtain significant suppression, analogous to TMS where a single session induces an immediate change in tinnitus perception while several sessions of low-frequency TMS induce prolonged effects [18, 43, 44]. Of course, special care such as screening of skin disease or abrasion, using a larger rubber electrode, and self-reporting of pain by the patient during stimulation are needed to elevate current to avoid skin burns [45].

Meanwhile, bifrontal tDCS for tinnitus patients have first been introduced by Vanneste et al. [38], based on previous studies reporting clinical benefits of bifrontal tDCS in treating major depression [46, 47], impulsiveness [48], and chronic pain [49, 50]. Bifrontal tDCS has been suggested to strengthen deficient inhibitory top-down mechanisms in tinnitus, making it possible to induce auditory sensory gating in the anterior cingulate cortex [51]. Also, Vanneste et al. has proposed that bifrontal tDCS may interfere with the emotional processing of tinnitus by modulating the cortico-subcortical and corticocortical pathways, as DLPFC may have a dampening effect on the midbrain-dorsomedial thalamic pathway, as has been shown for the somatosensory system [38]. A recent study using quantitative electroencephalography has indicated that responders to bifrontal tDCS seem to differ in resting state brain activity compared to nonresponders in the right auditory cortex and parahippocampal area [39].

In contrast to LTA-tDCS, bifrontal tDCS studies have demonstrated that switched polarity of the electrode pads was also effective for tinnitus management. That is, bifrontal tDCS placing the anodal electrode on the right DLPFC and the cathodal electrode on left DLPFC also could improve tinnitus intensity and tinnitus-related distress [38]. Moreover, the same group has suggested that left-anode bifrontal tDCS predominantly modulated tinnitus-related depression while right-anode bifrontal tDCS predominantly improved tinnitus-related anxiety [36]. This could be related to the prefrontal lateralization of tinnitus distress-related brain activity and tinnitus depression-related brain activity: whereas tinnitus-related distress is related to right lateralized alpha activity in the subgenual anterior cingulate cortex extending into the orbitofrontal/frontopolar areas, tinnitus-related depression is related to the same oscillation frequency but left lateralized in similar brain areas [52]. These lateralized affective results of bifrontal tDCS are in accordance with similar findings in previous tDCS studies on psychiatric disorders [53], and also show that, unlike LTA-tDCS, bifrontal tDCS polarity may be adjusted to tinnitus patients' primary combined psychiatric symptoms. However, future studies directly comparing LTA-tDCS and bifrontal tDCS are needed to further confirm the current preliminary conclusions.

## 5. Conclusions

At this stage, the efficacy of tDCS in treating tinnitus patients cannot be confirmed because only 2 RCTs were eligible for meta-analysis. However, not only the 2 studies included yielded significant improvement in tinnitus intensity by active tDCS as compared with sham tDCS, but also all the studies included in the current systemic review demonstrated significant improvement of tinnitus intensity. Therefore, although supported by a limited number of studies, tDCS is a promising tool for tinnitus management, meriting further research. 

No standard treatment protocol of tDCS in tinnitus management is available at the moment. Future RCTs in a large series of patients regarding the efficacy of tDCS as well as the comparison between LTA-tDCS and bifrontal tDCS are recommended to further validate the role of tDCS and to set up a standard treatment protocol.

## Figures and Tables

**Table 1:** Summary of the 6 studies that were included in the current study.

Study	Group	*n*	Anode	Cathode	Current, mA	Duration	Number of sessions	Measure	Mean percentage reduction	Percentage responders	Summary
Randomized controlled studies											

Fregni et al. (2006) [30]	Active, anodal	7	LTA	R SO	1	3 min	2	VAS tinnitus reduction scale (0–4)	30.36%	42.90%	
	Active, cathodal	7	R SO	LTA		3 min	2		0	0	
	Sham	7	LTA	R SO		5 sec	2		0	0	
Faber et al. (2011) [36]	Active, anode left	8	L DLPFC	R DLPFC	1.5	20 min	3	VAS intensity, VAS distress	27.80%		8 weeks' wash-out period between active and sham sessions changes measured directly after tDCS
	Active, anode right	7	R DLPFC	L DLPFC	1.5	20 min	3		0.00%	NA	
	Sham	15			1.5	30 sec	3		4.50%		
Garin et al. (2011) [37]	Active, anode left	20	L LTA	R VLPFC	1	20 min	1	VAS tinnitus reduction scale (0–4)	9.13%	35%	2 weeks' wash-out period between active and sham sessions
	Active, anode right	20	R VLPFC	L LTA	1	20 min	1			30%	
	Sham	20	L LTA	R VLPFC	110 *μ*A	20 min	1		0		
			(or vice versa)							

Open label studies											

Vanneste et al. (2010) [38]	Active, anode left	448	L DLPFC	R DLPFC	1.5	20 min	1	VAS intensity, VAS distress	0	0.00%	Changes measured directly after tDCS
	Active, anode right	30	R DLPFC	L DLPFC			1		7.95%	29.90%	
Vanneste et al. (2011) [39]	Active, anode right	45	R DLPFC	L DLPFC	1.5	20 min	1		14.00%	46.67%	changes measured directly after tDCS
Frank et al. (2012) [40]	Active, anode right	32	R DLPFC	L DLPFC	1.5	30 min	6	THI, TQ, BDI, CGI	NA	40.63%	

S.D.: standard deviation; LTA: left temporal area; SO: supraorbital; min: minutes; sec: seconds; VAS: visual analogue scale; DLPFC: dorsolateral prefrontal cortex; NA: not available; THI: tinnitus handicap inventory; TQ: tinnitus questionnaire; BDI: Beck depression inventory; CGI: clinical global impression scale.

**Table 2:** Forest plot of effect sizes (Hedges' *g*) for active versus sham transcranial direct current stimulation. CI, confidence interval.